# Physicochemical Characteristics and Microbial Communities in Gochujang, a Traditional Korean Fermented Hot Pepper Paste

**DOI:** 10.3389/fmicb.2020.620478

**Published:** 2021-01-18

**Authors:** Jung-A Ryu, Eiseul Kim, Mi-Ju Kim, Shinyoung Lee, Sung-Ran Yoon, Jung-gi Ryu, Hae-Yeong Kim

**Affiliations:** ^1^Gyeongsangbuk-do Agricultural Research and Extension Services, Daegu, South Korea; ^2^Department of Horticulture, Kyungpook National University, Daegu, South Korea; ^3^Institute of Life Sciences and Resources and Department of Food Science and Biotechnology, Kyung Hee University, Yongin, South Korea

**Keywords:** gochujang, physicochemical characteristics, high-throughput sequencing, microbial community, *Bacillus*, *Zygosaccharomyces*

## Abstract

Gochujang is a Korean fermented hot pepper paste beneficial to human health by providing various nutrients. In this study, its physicochemical characteristics were identified, and its microbial communities were analyzed by high-throughput sequencing. The interrelationship between physicochemical characteristics and microbial composition was investigated to reveal the properties of gochujang before and after fermentation. After fermentation, all samples showed decreased salt concentration, pH, and reducing sugar content, while the acidity and amino-type nitrogen increased. The water content, salt concentration, amino-type nitrogen, and reducing sugar differed according to the batches of samples. *Bacillus, Aerosakkonema*, and *Enterococcus* were identified as the predominant bacterial genera. Furthermore, *Aerosakkonema* was the most abundant genus before fermentation; however, it was replaced by *Bacillus* as it decreased after fermentation. For the fungi, *Aspergillus* dominated before fermentation, whereas *Zygosaccharomyces* and *Millerozyma* dominated after fermentation. The high level of amino-type nitrogen in gochujang was related to the relative abundance of *B*. *haynesii/B. licheniformis* before fermentation. Additionally, the high abundance of *Z. rouxii* after fermentation was related to the flavor of gochujang. This comprehensive analysis of the microbial community associated with the physicochemical properties of gochujang could help in understanding the factors affecting the quality of the product.

## Introduction

Gochujang is a traditional Korean fermented food made from fermented soybean (meju), red pepper powder, and glutinous rice. It is a popular flavoring condiment in East Asian countries along with doenjang (Korea), doubanjiang (China), and miso (Japan) (Baek et al., 2019; Chun et al., 2020). Gochujang creates a unique flavor by combining the spicy taste of red pepper powder with the sweetness and savory taste produced by the hydrolysis of carbohydrates and soybean proteins (Jang et al., 2011). The condiment has been highlighted as a nutrient source providing capsaicin, amino acids, soyasaponins, and soy isoflavones and as a beneficial food, improving human health owing to its anti-obesity, anti-cancer, anti-tumor, and anti-oxidant properties (Park et al., 2001; Hwang et al., 2017; Chun et al., 2020).

The traditional gochujang manufacturing process induces saccharification caused by heating glutinous rice and malt, and it is fermented for one or two years by adding meju, red pepper powder, and salt depending on the desired characteristics of taste and flavor (Jang et al., 2011). Meju is added to gochujang as a starter, and the quality of gochujang is affected by the composition of the microbial community of meju (Jang et al., 2011). In this regard, *B. subtilis* and *B*. *licheniformis* were the most important bacteria in meju, and the fungi were *Aspergillus, Mucor, Pichia*, and *Debaryomyces* (Jung et al., 2014). The relative abundance of these microorganisms in the ingredients and their interactions play important roles in determining the quality of the final fermented food product (gochujang) (Jiang et al., 2020). Microbial communities can change the compositions of metabolites and enzymes during fermentation by modifying their physicochemical characteristics such as pH, acidity, and level of amino-type nitrogen. Therefore, it is important to analyze the microbial communities and their physicochemical characteristics and their interrelationships, as a basis for improving the quality of fermented foods (Jiang et al., 2020).

Studies have been conducted to determine the composition of microbial communities and the characteristics of fermented foods such as kimchi and doubanjiang (Zhang et al., 2020; Kim et al., 2021). However, studies on the relationships between the microbial communities and the physicochemical characteristics of gochujang are few compared to other fermented foods. In particular, traditional fermented gochujang is produced by natural fermentation without starter cultures, leading to the formation of diverse microbial communities (Jung et al., 2016). Thus, fewer ecological niches resulting from high species richness may a result of the production of undesirable metabolites such as toxins and biogenic amines (Jung et al., 2016).

High-throughput sequencing technology based on 16S rRNA gene or internal transcribed spacer (ITS) sequences has been applied to confirm microbial communities in foods because it enables more accurate identification of complex microbial communities compared with other conventional microbiological methods such as denaturing gradient gel electrophoresis and other culture-independent based approaches (Jung et al., 2016; Zhang et al., 2020). In this study, the composition of microbial communities was analyzed by high-throughput sequencing both before and after the fermentation of gochujang samples obtained from various regions in Korea. In addition, the physicochemical changes related to the flavor of gochujang, including amino-type nitrogen and reducing sugars, were investigated to evaluate the correlation between microbial communities and chemical factors. In particular, the resultant data are expected to increase our knowledge of gochujang by finding microbial communities related to chemical features such as amino-type nitrogen that contribute to improving the quality of gochujang.

## Materials and Methods

### Sampling

Gochujang samples obtained from four different regions of Korea were collected before (0 day) and after fermentation (12 months), respectively. The samples were selected Uljin (UJ) and Yeongju (YJ), the northern regions of Gyeongsangbuk-do in Korea, and Goryeong (GR) and Gyeongsan (GS), the southern regions, it was confirmed whether regional differences in climatic conditions affect the characteristics of gochujang. All samples were small-scale farmer-type traditional gochujang produced without a starter culture and processed in the same way according to the traditional manufacturing method. The gochujang samples selected for this study were certified as traditional foods products by a Korean grandmaster. After collection, the gochujang samples were immediately transferred to the laboratory and stored at 4°C in an ice cooler for the subsequent determination of quality characteristics.

### Measurement of Physicochemical Characteristics

#### Measurement of Water Content, Salt Concentration, and Color Characteristics

The water content of gochujang was measured by weighing 2 g samples, then drying them in a drying oven at 105°C to a constant dry weight according to the modified AOAC method (AOAC, 2005). To measure the salt concentration, 2.5 g of each sample was added 47.5 mL of boiled water and then extracted using a shaking incubator at 500 rpm for 1 h. After filtering through filter paper, 5% K_2_CrO_4_ indicator was added to 10 mL of the filtrate, and it was titrated with 0.1 N AgNO_3_ until it changed from yellow to brownish–red. The color characteristics of gochujang were measured with a colorimeter (JS555, Color techno system Co., Tokyo, Japan) to produce L (lightness), a-value (redness), and b-value (yellowness).

#### Measurement of pH and Acidity

For the pH and acidity measurement, 9 g of each sample was added to 47.5 mL distilled water, blended in an electric blender (Polytron PT-MR 2100, Kinematica AG, Lucerne, Switzerland), and centrifuged at 6,200 × *g* for 10 min. The supernatant was then filtered through filter paper, and the filtrate of each sample was measured using a pH meter (Corning 340, Corning Co., New York, NY, USA). The acidity was measured with 0.1 N NaOH, yielding a titration endpoint at pH 8.3, and the percentage of lactic acid (%, v/v) was determined as the measured volume of 0.1 N NaOH.

#### Measurement of Amino-Type Nitrogen and Reducing Sugars

The amino-type nitrogen was measured according to the formol titration method (AOAC, 1990). The filtrate was added to a formalin solution and titrated with 0.1 N NaOH to a pH of 8.3. The measured volume of 0.1 N NaOH was calculated as the amino-type nitrogen content (mg %). The reducing sugar was measured according to the 3,5-dinitrosalicylic acid (DNS) method (Gil et al., 2017). The filtrate was added to the DNS reagent, and the mixture was heated in a water bath at 100°C for 5 min. After cooling to room temperature, the absorbance of the mixture was measured using a spectrophotometer (Optizen 2120UV, Mecasys Co. Ltd., Daejeon, Republic of Korea) at 550 nm. The reducing sugar content was calculated based on a standard curve prepared with predetermined glucose.

#### Measurement of Viable Cell Counts

Viable cell counts were determined according to a previous study (Gil et al., 2017). Briefly, 1 g of the sample was homogenized in 9 mL of 0.85% (w/v) saline solution, and the homogenized sample was serially diluted. The diluted samples were spread on tryptic soy agar (BD Difco, Detroit, MI, USA), Lactobacilli MRS agar (BD Difco), and CHROMagar *Bacillus cereus* (CHROMagar, Paris, France) for the enumeration of total viable bacteria, lactic acid bacteria, and *B. cereus*. Additionally, yeast and mold petrifilm (YM) (3M, St, Paul, MN, USA) was used for the enumeration of yeast and mold. Coliform was counted using petrifilm coliform count plate (CC) (3M) and incubated at 37°C for 24 h. Total viable bacteria, *B*. *cereus*, and yeast and mold were incubated aerobically at 30°C for 24, 24–48, and 72–96 h, respectively. Lactic acid bacteria were incubated anaerobically at 30°C for 48 h and the cell count was calculated as colony forming unit per gram (CFU/g).

### Illumina MiSeq Sequencing on 16S rRNA and ITS Regions

The total genomic DNA of gochujang was extracted using PowerMax® Soil DNA Isolation Kit (MO BIO, Carlsbad, CA, USA) according to the manufacturer's instructions. The DNA quality and concentration were confirmed using a PicoGreen kit (Invitrogen, Carlsbad, CA, USA). The bacterial community was amplified with the universal primer pair 341F (5'-adaptor-CCT ACG GGN GGC WGC AG-3') and 805R (5'-adaptor-GAC TAC HVG GGT ATC TAA TCC-3') targeting the V3-V4 region of the 16S rRNA gene sequence. The fungi community was amplified with the universal primer pair ITS3 (5'-adaptor-GCA TCG ATG AAG AAC GCA GC-3') and ITS4 (5'-adaptor-GTC CTC CGC TTA TTG ATA TGC-3') targeting the ITS regions. The library was prepared according to the manufacturer's instruction and sequencing on an Illumina MiSeq (Illumina, San Diego, CA, USA) system.

The raw sequences were sorted based on barcode sequences, and the adapter and barcode sequences were removed. Additionally, sequences involving ambiguous bases (N), sequences <450 bp (bacteria) or 460 bp (fungi), chimeric sequences, and other sequence errors were filtered and trimmed to obtain clean sequence data. The processed sequence reads were normalized, and microbial communities in the gochujang samples were analyzed by QIIME v.2 (Caporaso et al., 2010; Bokulich et al., 2018) based on the SILVA rRNA database (Quast et al., 2012). Their diversity indices, such as the operational taxonomic unit (OTU) and beta diversity (weighted and unweighted UniFrac), were calculated using the QIIME v. 2 pipeline. Wilcoxon rank-sum test (unpaired) was used to compare microbial communities before and after fermentation, and *p* < 0.05 was determined to be statistically significant. Hierarchical clustering was analyzed based on the beta diversity distance matrix, and the tree structure was constructed by the unweighted pair group method with arithmetic mean (UPGMA). The principal coordinate analysis (PCoA) based on weighted UniFrac analysis was performed to visualize microbial community in gochujang. The raw sequencing data were deposited in the Sequence Read Archive (SRA) of National Center for Biotechnology Information (NCBI) under the accession number PRJNA673648.

### Statistical Analysis

All experiments for physicochemical properties were performed in triplicate, and the statistical analysis of experimental values was performed using R version 4.0.2. In addition, significant differences (*p* < 0.05) between the mean values were determined by Duncan's multiple range test. Relationships between physicochemical properties and microbial community structures were analyzed by Pearson correlation regression analyses implemented in R (Santiyanont et al., 2019).

## Results

### Physicochemical Characteristics

**Figure 1:**
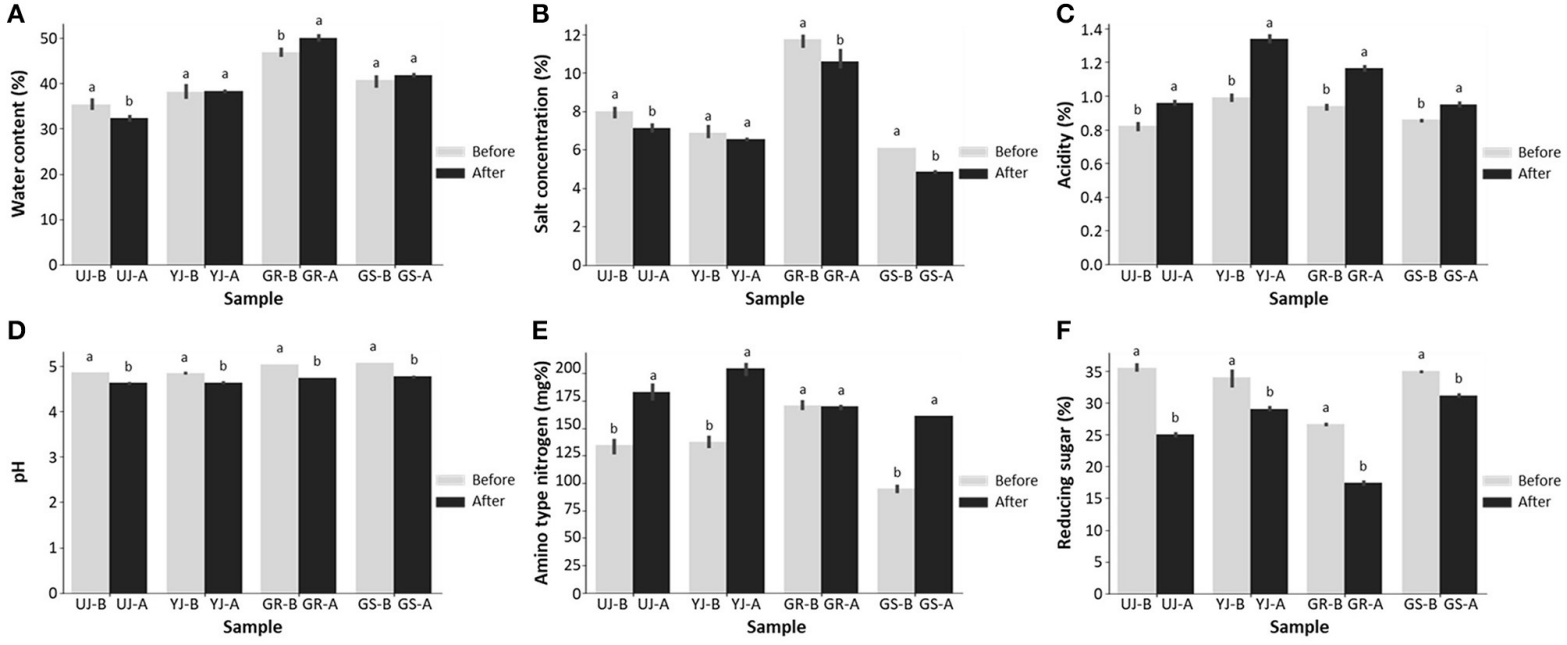
Differences before and after fermentation of **(A)** water content, **(B)** salt concentration, **(C)** acidity, **(D)** pH value, **(E)** amino-type nitrogen, and **(F)** reducing sugar of gochujang produced in four regions. Data values are indicated as the mean ± standard deviation. Values followed by different letters in the same sample are significantly different (*p* < 0.05).

All gochujang samples before and after fermentation were collected from various regions in Korea, and their physicochemical characteristics were compared. The water content of the GR sample increased slightly from 46.9 ± 0.81% to 50.1 ± 0.56% after fermentation, while the UJ sample decreased slightly from 35.4 ± 1.05% to 32.3 ± 0.52% after fermentation (Figure 1A). The YJ and GS samples did not show any difference in water content before and after fermentation. The water content of the GR sample (48.5%) and the GS sample (41.4%) were higher than the overall average (40.5%), and the remaining samples showed lower water content values than the average. The salt concentration in all gochujang samples decreased after fermentation. Similar to other fermented foods, it is presumed that the salt concentration decreased due to the release of water by osmosis of the ingredients (Lee et al., 2017; Liang et al., 2019). They were ~8.2 and 7.3%, before and after fermentation, respectively (Figure 1B). The GR sample showed a higher salt concentration than the overall averages before (11.7 ± 0.34%) and after (10.6 ± 0.53%) fermentation.

The acidity increased after fermentation (Figure 1C), and the pH value was decreased slightly in all samples (Figure 1D). In particular, the acidity of the YJ and GR samples increased significantly from about 0.96% to about 1.3% after fermentation. The overall pH values of the UJ and YJ samples were slightly lower than the other samples, while the acidity of the YJ and GR samples was slightly higher than other samples. Amino-type nitrogen is closely related to the quality of gochujang and is an index to determine the degree of fermentation (Baek et al., 2019; Chun et al., 2020). The content of amino-type nitrogen increased during the fermentation, peaked and after fermentation at a concentration of 182.9 mg%, which was ~1.6 times higher than before fermentation, except for the GR sample (Figure 1E). The GR sample did not show a difference in amino-type nitrogen before and after fermentation. Significant differences in reducing sugar content were observed among the samples; before fermentation, the values ranged from 26.6 to 35.4%, but after fermentation, the range was from 17.4 to 31.1% (Figure 1F). In particular, the reducing sugar content of the GR sample, the lowest of all samples both before (26.6 ± 0.13%) and after (17.4 ± 0.21%) fermentation.

**Figure 2:**
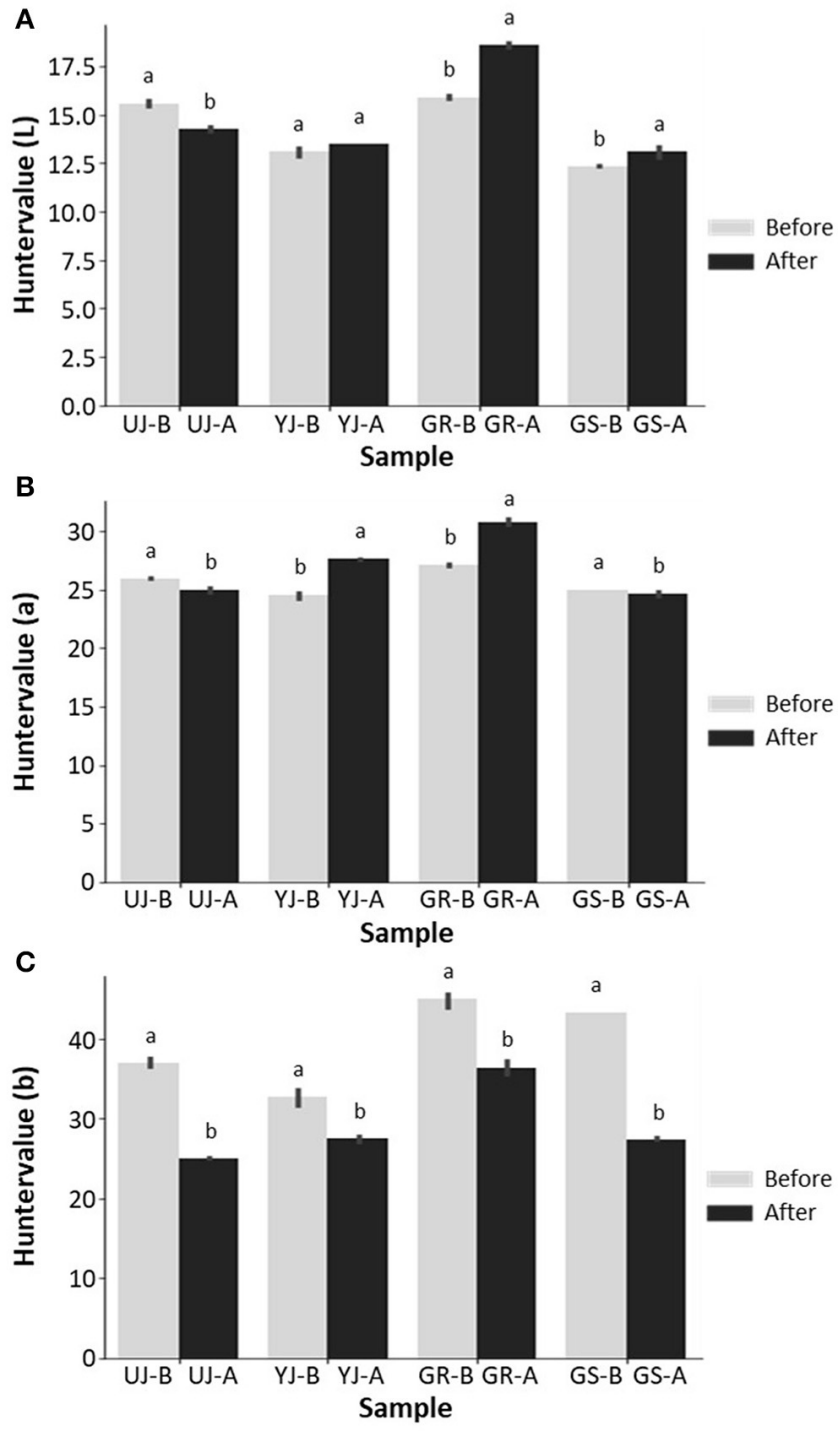
Color parameters of **(A)** L (lightness), **(B)** a (redness), and **(C)** b (yellowness) of gochujang produced in four regions. Data values are indicated as the mean ± standard deviation. Values followed by different letters in the same sample are significantly different (*p* < 0.05).

The color of gochujang was correlated with the consumer's preference for connecting this criterion to quality evaluation. The lightness of the gochujang color increased slightly from 13.8 to 15.1% after fermentation except for the UJ sample. The UJ sample decreased slightly from 15.6 to 14.3% after fermentation (Figure 2A). The redness decreased slightly in the UJ and GS samples and increased slightly in the remaining samples (Figure 2B). Yellowness decreased rapidly after fermentation in all samples, and among the colors, it showed the greatest change before and after fermentation (Figure 2C). The lightness, redness, and yellowness values of the GR sample were higher than those of the other samples.

### Changes in Viable Cell Counts

Overall, the number of viable cells showed variations between batches and before and after fermentation. The total viable bacterial cell count for the samples before fermentation was 7.14–8.02 log CFU/g. The total viable cells decreased slightly in the YJ and GS samples after fermentation, while they increased in the GR sample (Table 1). The UJ sample showed no significant difference in total viable cell count before and after fermentation. The number of lactic acid bacteria decreased in most samples except for the GS sample, in which it increased after fermentation. Differences in the yeast count were observed among all the samples; the yeast cell count in gochujang before fermentation was in the range of 2.81–5.39 log CFU/g. Coliform did not appear in all samples similar to the previous study (Baek et al., 2019).

**Table 1:** Viable cell counts of gochujang before and after fermentation produced in four regions.

**Items**	**Period**	**Gochujang (Log CFU/g)**^****a****^			
		**UJ**	**YJ**	**GR**	**GS**
Aerobic bacteria	Before	7.74 ± 0.04^Ab^	8.02 ± 0.01^Ba^	7.85 ± 0.05^Bab^	7.14 ± 0.21^Bc^
	After	7.8 ± 0.03^Ac^	7.9 ± 0.03^Ab^	8.1 ± 0.03^Aa^	6.7 ± 0.03^Ad^
Coliform	Before	0.0 ± 0.00	0.0 ± 0.00	0.0 ± 0.00	0.0 ± 0.00
	After	0.0 ± 0.00	0.0 ± 0.00	0.0 ± 0.00	0.0 ± 0.00
Yeast	Before	5.39 ± 0.03^Aa^	4.55 ± 0.05^Ab^	2.81 ± 0.13^Bd^	3.28 ± 0.11^Bc^
	After	4.2 ± 0.13^Bb^	4.1 ± 0.17^Bb^	4.7 ± 0.13^Aa^	4.1 ± 0.14^Ab^
*B. cereus*	Before	1.52 ± 0.07^Ad^	2.16 ± 0.05^Bb^	4.70 ± 0.03^Ba^	1.63 ± 0.06^Ac^
	After	0.4 ± 0.75^Ac^	2.4 ± 0.05^Ab^	4.8 ± 0.01^Aa^	0.9 ± 0.81^Ac^
Lactic acid bacteria	Before	6.9 ± 0.04^Aa^	4.6 ± 0.07^Ad^	6.3 ± 0.06^Ab^	4.8 ± 0.02^Bc^
	After	5.6 ± 0.06^Ba^	4.4 ± 0.05^Bc^	5.2 ± 0.09^Bb^	5.2 ± 0.14^Ab^

a *Data values indicated as the mean ± standard deviation of three replications. Values followed by different letters in the same region (A–B) or same fermentation time (a–d) are significantly different (p < 0.05)*.

### Microbial Communities

**Figure 3:**
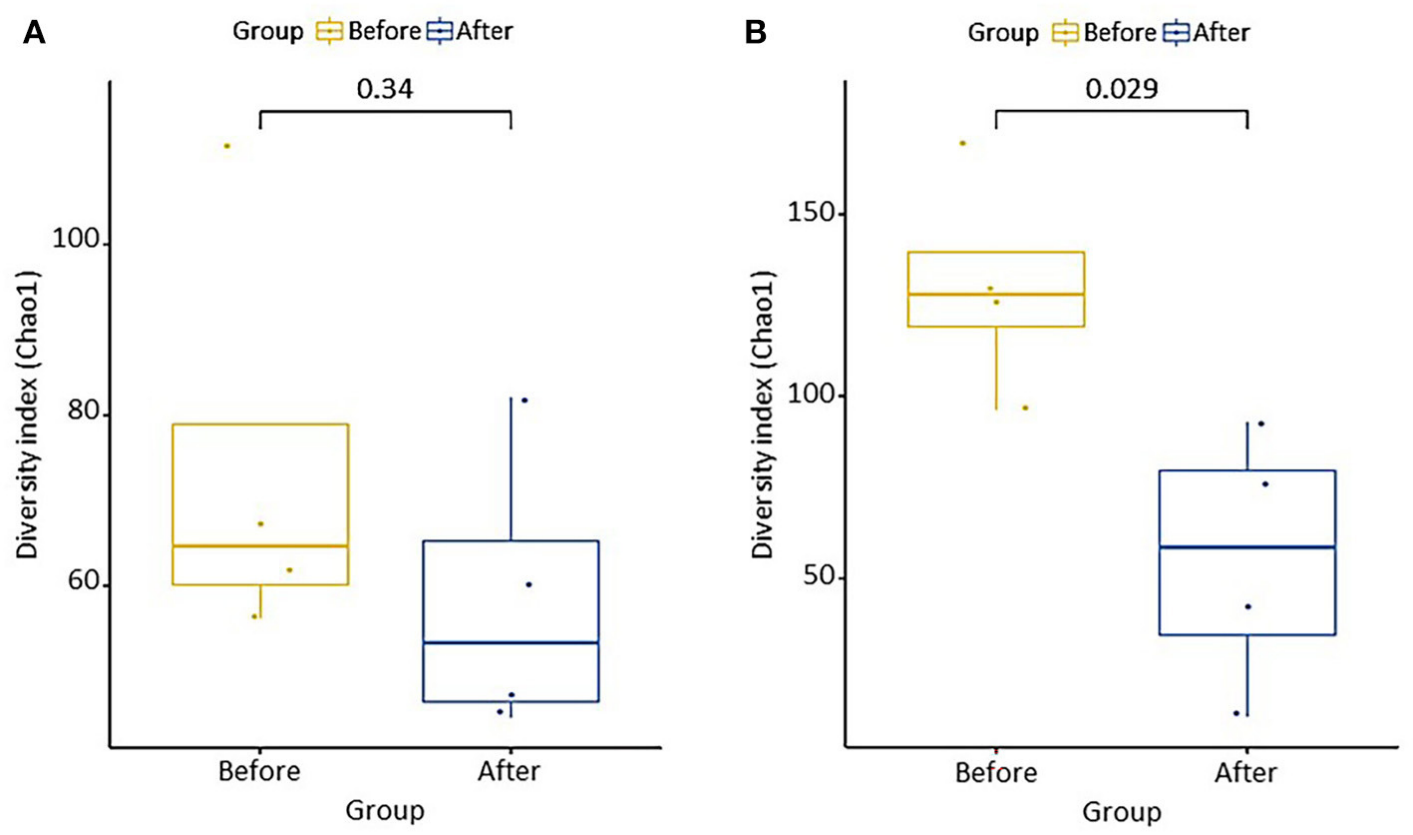
Chao1 richness boxplot showing difference of **(A)** bacterial and **(B)** fungal microbial communities before and after fermentation. Group difference was determined Wilcoxon rank-sum test.

**Figure 4:**
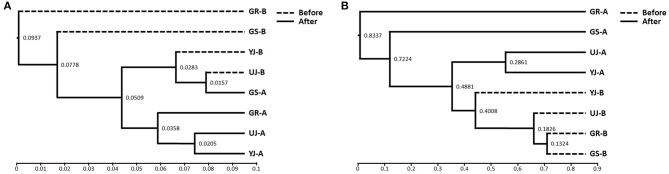
Hierarchical clustering analysis of **(A)** bacterial and **(B)** fungi communities in gochujang samples based on the unweighted pair group method with arithmetic mean (UPGMA).

**Figure 5:**
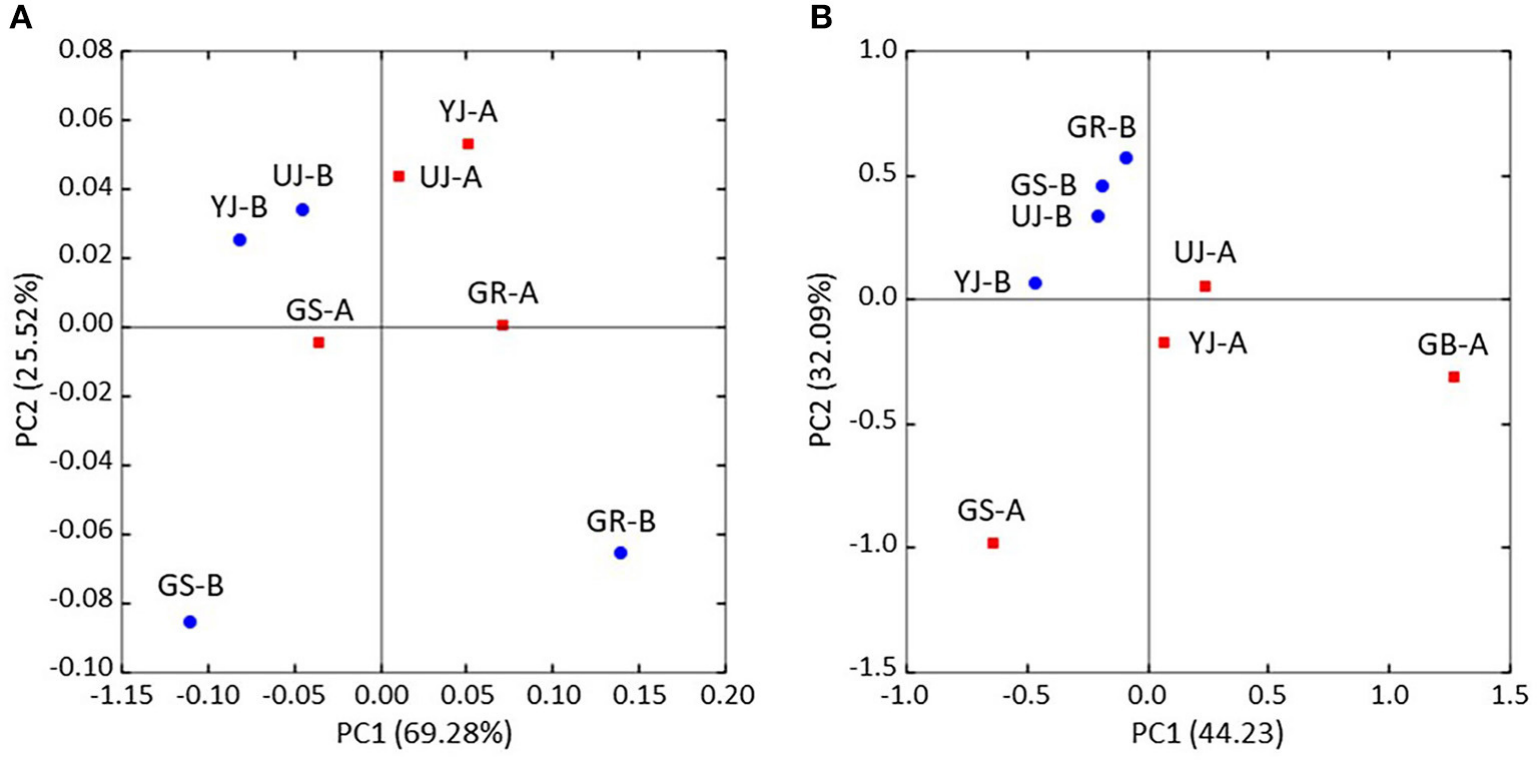
Principal coordinate analysis (PCoA) of **(A)** bacterial and **(B)** fungi communities in gochujang samples based on the weighted UniFrac metrics. Blue and red symbols represent samples before and after fermentation, respectively.

The differences in microbial communities before and after fermentation were analyzed. The richness (Chao1) in the bacterial community before and after fermentation was not significant (*p* = 0.343), and the fungal community was significantly different (*p* = 0.029) (Figure 3). On the other hand, diversity (Shannon) was not significant for both bacterial (*p* = 0.486) and fungal (*p* = 0.114) communities. *Zygosaccharomyces* (*p* = 0.029) and *A. oryzae* (*p* = 0.029) showed significant differences before and after fermentation at the genus and species level, respectively. The diversity index was higher in the fungal community before fermentation compared to the community after fermentation. The hierarchical clustering and PCoA were analyzed to understand the similarities and differences in the composition of microorganisms according to the batches of samples, which revealed four different clusters (Figure 4). The GR samples showed a distinctly different bacterial composition before fermentation compared to the other samples (Figure 4A), and its fungal composition after fermentation was also most distinctly different from the other samples (Figure 4B). PCoA based on weighted UniFrac analysis also indicated a structural difference between samples (Figure 5).

**Figure 6:**
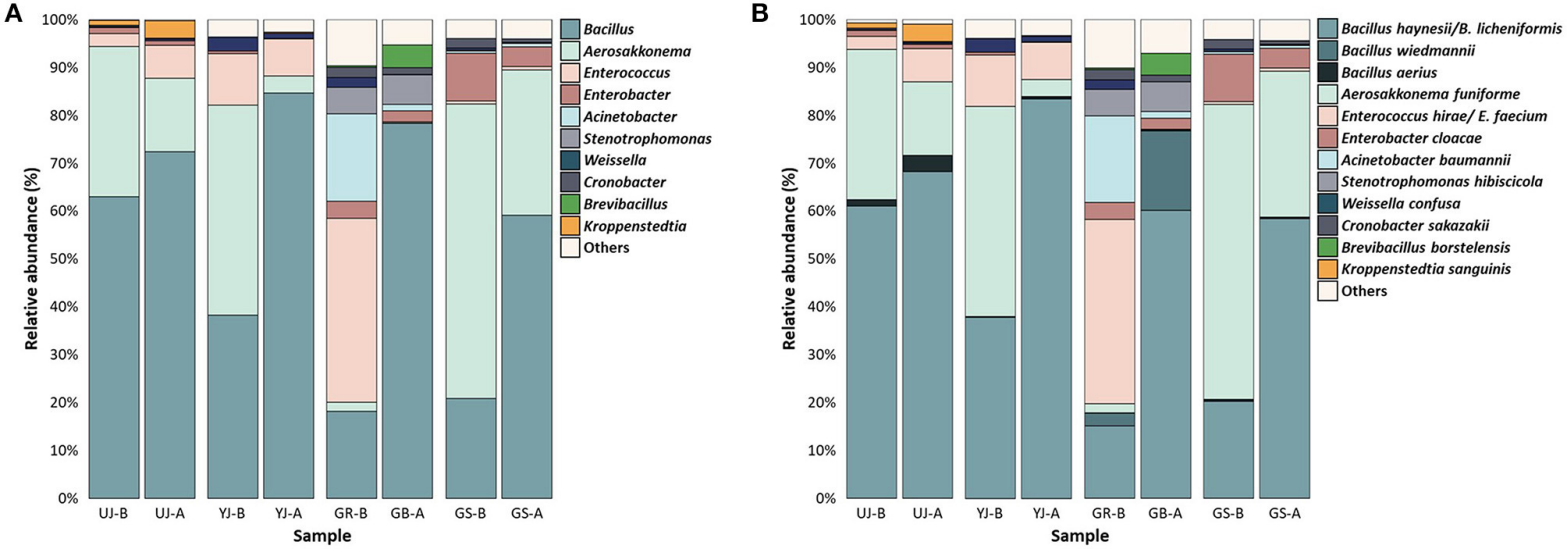
Bacterial composition in the gochujang samples before and after fermentation. Data portray **(A)** genus and **(B)** species level of 16S rRNA gene sequences. The “others” are composed of the genera or species with a prevalence of <0.5% of the total reads in each (all) sample. “-B” and “-A” mean before and after fermentation, respectively.

To identify the microbial communities, the OTUs from the 16S rRNA and ITS sequencing reads were classified at the genus and species levels. Since this study assigned OTUs using the V3-V4 region, it may not provide sufficient resolution to the species level, but it can be assigned to the genus level. In all samples, at the genus level in the bacterial communities, *Bacillus* (54.70%), *Aerosakkonema* (23.75%), and *Enterococcus* (8.50%) were predominant, followed by *Enterobacter, Acinetobacter*, and *Stenotrophomonas* (Figure 6A). In all samples, *Aerosakkonema* decreased after fermentation, and instead, *Bacillus* was dominant after fermentation. *Aerosakkonema*, known as one of the gas-vacuolated oscillatorioid, is a microorganism distributed in the environment such as reservoirs (Thu et al., 2012). This is similar to a previous study in which *Aerosakkonema* abundantly existed before fermentation in radish kimchi (Mannaa et al., 2019). Overall, the presence or absence of fermentation did not influence the bacterial communities of gochujang, but the GR sample before fermentation showed a difference from other samples in hierarchical clustering analysis. *Enterococcus* and *Acinetobacter* were dominant in the GR sample, and *Bacillus* and *Aerosakkonema* were present in low relative abundance compared to other samples. At the species level, *B*. *haynesii/B. licheniformis* (51.01%), *A*. *funiforme* (23.75%), and *E*. *hirae/E. faecium* (8.49%) were dominant in all the samples (Figure 6B). In all samples, *B*. *haynesii/B. licheniformis* was observed in relatively higher abundance after fermentation. In all samples, *A*. *funiforme* almost disappeared after fermentation and was replaced by *B*. *haynesii/B. licheniformis*.

**Figure 7:**
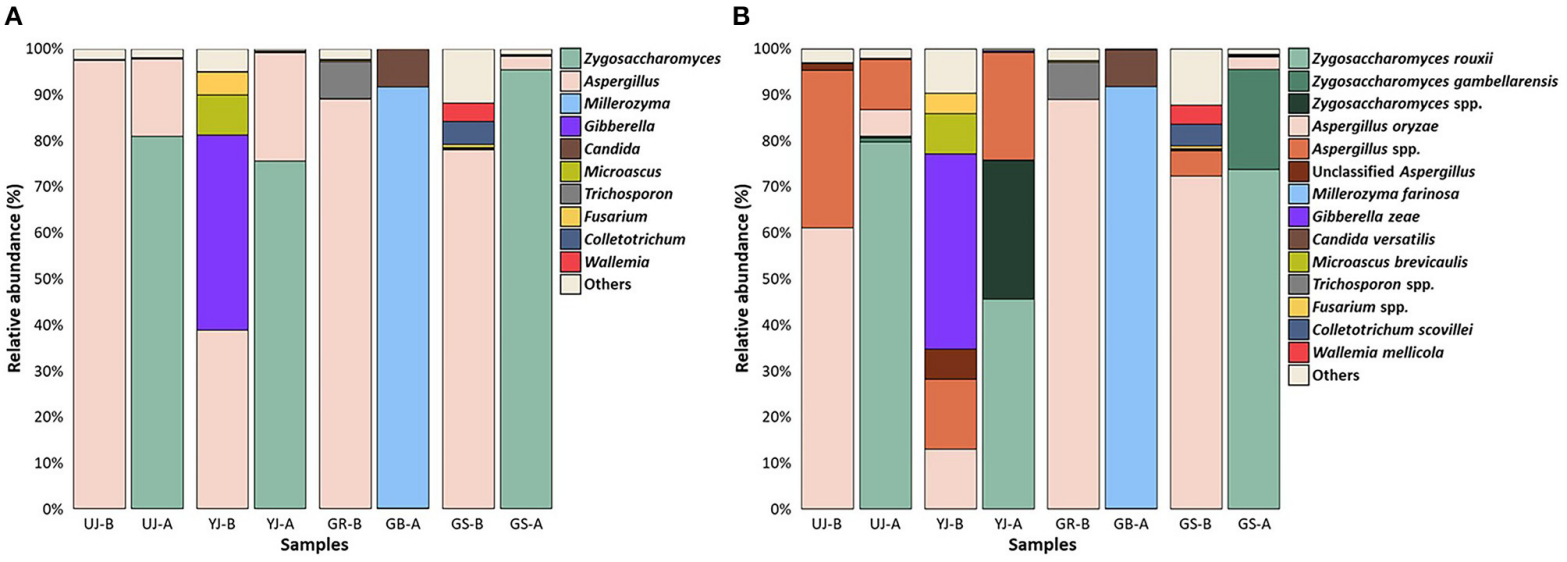
Fungal composition in the gochujang samples before and after fermentation. Data portray **(A)** genus and **(B)** species level of internal transcribed spacer sequences. The “others” are composed of the genera or species with a prevalence of <0.5% of the total reads in each sample.

For fungal communities at the genus level, *Zygosaccharomyces* (36.32%), *Aspergillus* (33.68%), *Millerozyma* (18.97%), and *Gibberella* (3.75%) were predominant in all samples, followed by *Candida, Microascus*, and *Trichosporon* (Figure 7A). In most samples, *Aspergillus* was observed at relatively high abundance before fermentation but disappeared and was replaced by *Zygosaccharomyces* after fermentation. However, the YJ and GR samples, before and after fermentation, respectively, showed different microbial communities from the other samples. In the YJ sample, *Aspergillus* was present before fermentation, unlike in the other samples, and *Gibberella* was also present at a relatively high abundance. After fermentation, the GR sample was dominated by *Millerozyma*, not *Zygosaccharomyces*. At the species level, *Z*. *rouxii, A. oryzae, M*. *farinosa*, and *G*. *zeae* were dominant in all samples (Figure 7B).

Correlation between physicochemical properties and relative abundance of bacterial and fungal was analyzed. *Zygosaccharomyces* was a fungus having a significant correlation with salt concentration after fermentation (Pearson coefficient *r* = −0.98, *p* = 0.018). Also, this fungus was significantly correlated with the difference before and after fermentation of amino-type nitrogen (*r* = 0.98, *p* = 0.018). *Bacillus* was the only bacteria having a correlation with amino-type nitrogen content (*r* = 0.67, *p* = 0.077). Other dominant bacteria, such as *Aerosakkonema* and *Enterococcus*, did not show significant correlations with amino-type nitrogen content (*r* < 0.16, *p* > 0.704).

## Discussion

The formation of specific nutrients and flavors in fermented foods is usually influenced by the microbial communities (Du et al., 2019; Jiang et al., 2020). Few studies have been performed on the microbial communities during the fermentation period of gochujang compared to other traditional Korean fermented foods, such as kimchi or doenjang (Chun et al., 2020; Kim et al., 2021). Thus, using high-throughput sequencing methods, this study revealed the differences in the microbial communities of gochujang according to the batches of samples and the presence or absence of fermentation. Furthermore, we investigated the effect of the bacterial composition on the quality of gochujang. As a result, the predominant bacterial and fungal genera of gochujang were determined. However, the relative abundance of bacteria and fungi was slightly different due to the batch-to-batch differences, which reflected the effects of the physicochemical environments such as salt concentration and amino-type nitrogen on the microbial communities.

Overall, the dominant bacteria genera in gochujang were *Bacillus, Aerosakkonema*, and *Enterococcus*, and the fungal genera were *Zygosaccharomyces, Aspergillus*, and *Millerozyma*. These are similar to the microbial communities of doubanjiang, a traditional Chinese food fermented with red pepper and meju (Zhang et al., 2020). The certification system for the quality of traditional foods in Korea stipulates that the water content of gochujang and amino-type nitrogen after fermentation should be <50% and more than 160 mg%, respectively (Baek et al., 2019). According to this standard, the water content and amino-type nitrogen of all samples used in this study met the regulation. In general, the water content was comparable to doubanjiang (Zhang et al., 2020), one of the Chinese traditional fermented foods, but lower than doenjang (Shim et al., 2016) and cheonggukjang, which are Korean fermented foods (Gil et al., 2016).

According to the hierarchical clustering analysis based on UPGMA, the microbial communities in all the gochujang samples showed similar patterns before and after fermentation, except for the GR sample. Before fermentation, the GR sample was observed to have a relatively higher abundance of *Enterococcus* than other samples, which might have originated from meju, one of the main ingredients of gochujang. Similarly, *Enterococcus* was identified as the most dominant bacteria on the exterior of fermented meju (Jung et al., 2014). The GR sample that showed the highest concentration of salt compared to the other samples had a relatively low abundance of *Bacillus* before fermentation. This may be because this bacterial genus does not grow well under high salt conditions (Chun et al., 2020). In addition, this sample was dominated by fungi *Millerozyma* rather than of *Zygosaccharomyces* after fermentation. According to the previous study, it was reported that *M*. *farinosa* appeared similar to *Z*. *rouxii* in the late period of mash fermentation (Wei et al., 2013); these results were consistent with our results. *Z. rouxii* is a highly osmophilic fungus, and *M. farinosa* is a halophilic fungus (Buzzini et al., 2018). *M*. *farinosa* grows better in higher salt conditions than *Z. rouxii* (Dakal et al., 2014). Because of this, it is plausible that after fermentation, the GR sample was dominated by *M. farinosa* rather than *Z. rouxii*.

The pH and acidity of gochujang samples decreased and increased, respectively, after fermentation. The pH and acidity significantly changed the optimal growth condition for some microorganisms; thus, the relative abundance of *Enterobacter* decreased after fermentation compared to before fermentation. In contrast, *Bacillus*, which is acid resistant and exhibits a high level of environmental adaptability (Ren et al., 2019), was relatively abundant after fermentation compared to before fermentation. In particular, this genus, which is known to promote the formation of flavor in fermented food by secreting various enzymes (Jiang et al., 2020), was the most abundant in the YJ sample that showed the highest acidity after fermentation. In addition, the fungal genera, such as *Zygosaccharomyces*, which are known to be tolerant to weak acidity and low pH (Kuanyshev et al., 2016), dominated in all samples after fermentation except for the GR sample. *Z. rouxii* is a major fungus that contributes to the synthesis of many volatile components that add flavors in the late stages of fermentation of doubanjiang (Zhang et al., 2020). Moreover, *Z. rouxii* is a fungus widely applied in the soy sauce industry because it can improve volatile components using Maillard intermediates as precursors (Hayashida et al., 1999; Wah et al., 2013; Zhang et al., 2020). In this regard, this fungus may be a key microorganism associated with the flavor of gochujang.

It is known that microorganisms use reducing sugars for their nutrition, decomposing them into alcohol and CO_2_ to produce the components giving the unique flavors and tastes to fermented foods (Choi et al., 2018). Additionally, the amino-type nitrogen, which includes free amino acids formed by the action of proteases secreted by microorganisms during the fermentation of soybean products, is associated with the quality and flavor of gochujang (Lee et al., 2016; Kim et al., 2017). Amino-type nitrogen increased in soybean products after fermentation due to the continuous degradation of proteins by proteases. In this study, most of the gochujang samples showed increased amino-type nitrogen after fermentation, but there was no difference in amino-type nitrogen levels before and after fermentation in the GR sample. *Bacillus* species are bacteria capable of synthesizing many enzymes and organic acids, and they can produce strong proteases and amylases (Ren et al., 2019). Therefore, the previous study reported that the levels of amino-type nitrogen in soybean products inoculated with *B. licheniformis* at the beginning of fermentation doubled after fermentation (Yoo et al., 2000). The reason for the low concentration of amino-type nitrogen in the GR sample after fermentation might be that the protein in the sample was not sufficiently degraded due to the relatively lower abundance of *Bacillus* before fermentation. Also, as in the previous studies, it is assumed that amino acids produced through proteolysis were consumed by fungi such as *Candida* (Jeong et al., 2013; Li et al., 2017). *Tetragenococcus* is resistant to high salt concentration and produces diverse proteases to improve the tasty amino acid content in fermented foods (Du et al., 2019). Thus, this genus was found to positively affect the accumulation of amino acids in fermented foods such as fish sauce and doenjang (Jung et al., 2016; Du et al., 2019). However, in this study, *Tetragenococcus* was present at low abundance in all samples (0.16%), and therefore, *Bacillus* was more involved in increasing the amino-type nitrogen levels of gochujang than *Tetragenococcus*, unlike in other fermented soybean foods.

In conclusion, the composition of microbial communities was different according to the presence or absence of fermentation in gochujang and the batches. The relative abundance of *Bacillus* and *Zygosaccharomyces* was related to the amino-type nitrogen content, one of the factors determining the quality of gochujang. Before fermentation, the level of *B*. *haynesii/B. licheniformis* should be higher than *Enterococcus*, which will help to increase the amino-type nitrogen level in gochujang after fermentation. In addition, in order to increase the relative abundance of *Z. rouxii*, which is positively related to the quality and flavor of gochujang, high salt concentration should be avoided during gochujang production. These results could provide helpful information toward understanding some of the factors involved in the production process and improve the quality of gochujang.

## Data Availability Statement

The datasets presented in this study can be found in the NCBI repository, accession number PRJNA673648.

## Author Contributions

J-AR and H-YK designed the experiment. J-AR and S-RY performed the analysis of physicochemical characteristics. EK and M-JK analyzed the microbial communities. J-AR and EK prepared a draft manuscript. J-AR, EK, SL, and H-YK reviewed and edited the manuscript. All authors read and approved the final manuscript.

## Conflict of Interest

The authors declare that the research was conducted in the absence of any commercial or financial relationships that could be construed as a potential conflict of interest.
